# Investigating the Role of Ly6G^+^ Neutrophils in Incisional and Inflammatory Pain by Multidimensional Pain-Related Behavioral Assessments: Bridging the Translational Gap

**DOI:** 10.3389/fpain.2021.735838

**Published:** 2021-09-10

**Authors:** Daniel Segelcke, Bruno Pradier, Sylvia Reichl, Lukas C. Schäfer, Esther M. Pogatzki-Zahn

**Affiliations:** ^1^Department for Anesthesiology, Operative Intensive Care and Pain Medicine, University Hospital Muenster, Albert-Schweitzer-Campus 1, Muenster, Germany; ^2^Department of Anesthesiology, Perioperative Medicine, Paracelsus Medical University, Salzburg, Austria

**Keywords:** translational, behavior, pain, incision, inflammatory neutrophils

## Abstract

In recent years, preclinical pain research has failed to develop genuinely new analgesics for clinical use. This fact is reflected by a high number of patients, limited drug efficacy accompanied by side effects, and a long-term opioid intake. Two main aspects have been addressed, which hinder translation: the use of non-relevant pain models and a mismatch between pain-related outcomes in preclinical and clinical studies. Conversely, disease-specific pain models that mirror more closely the clinical situation and multidimensional behavioral outcome measures that objectively and reproducibly assess relevant pain-related symptoms in a preclinical setting could improve translation. Mechanistically, a matter of debate is the role of Ly6G^+^ neutrophil granulocytes (NGs) for pain. NGs are essential to eliminate pathogens and promote the wound healing process. For this purpose, there is a need to release various pro- and anti-inflammatory mediators, some of which could ameliorate or enhance pain. However, the contribution of NGs to different pain entities is contradictory for reflex-based tests, and completely unknown in the context of non-evoked pain (NEP) and movement-evoked pain (MEP). First, we combined withdrawal reflex-based assays with novel video-based assessments for NEP- and MEP-related behavior in two mouse pain models. The pain models utilized in this study were incision (INC) and pathogen/adjuvant-induced inflammation (CFA), translating well to postsurgical and inflammatory pain entities. Second, we depleted NGs and applied a set of behavioral assessments to investigate the role of NG migration in different pain modalities. Our comprehensive behavioral approach identified pain-related behaviors in mice that resemble (NEP) or differentiate (MEP) behavioral trajectories in comparison to mechanical and heat hypersensitivity, thereby indicating modality-dependent mechanisms. Further, we show that injury-induced accumulation of NGs minimally affects pain-related behaviors in both pain models. In conclusion, we report a novel assessment to detect NEP in mice after unilateral injuries using a more unbiased approach. Additionally, we are capable of detecting an antalgic gait for both pain entities with unique trajectories. The different trajectories between MEP and other pain modalities suggest that the underlying mechanisms differ. We further conclude that NGs play a subordinate role in pain-related behaviors in incisional and inflammatory pain.

## Introduction

Although preclinical pain research has increased our understanding of pathophysiological mechanisms related to pain, there is still no major forthcoming initiative in developing effective and safe analgesics. As a result, many patients suffer from acute and chronic pain as current analgesics have limited efficacy and side effects. One reason that hampers the development of new treatment options is a translational gap from basic science to the clinical situation (1, 2). For example, there is increasing evidence for pain entity-specific mechanisms; therefore, the development and use of preclinical pain models, which more precisely represent clinical situations, are highly needed (1, 3). Further, outcome assessments in preclinical and clinical research represent a significant mismatch and are the subject of current discussion in clinical and preclinical areas (1). From a historical perspective, evoked pain-related behaviors are assessed by determining withdrawal reactions to external applied mechanical and thermal stimuli to the hindpaw of rodents. However, these assessments [still used in most preclinical pain studies (2, 4, 5)] are not only prone to experimental bias (6); they also encompass mainly the somatosensory (spinal reflex) pathways and miss the complex cognitive and emotional as well as voluntary components of pain. In contrast, multidimensional behavioral outcome measures in preclinical settings presumably capturing clinically relevant symptoms might improve translation (1, 7–9).

In the field of multidimensional pain-related behavioral testing, there have been developments in the past two decades, which, on the one hand, addresses functional components, such as pain-related gait changes, and on the other hand, non-evoked pain- (NEP-) related behavior assessments (7, 8, 10). In addition, video-based methods for assessing pain-related outcomes have been developed to reduce experimental bias and increase transparency and study quality (7, 8, 11). The rise of video-based methods opens up a way to refine methodologies on the same animals, ultimately reducing animal numbers regarding the replacement, reduction, and refinement (3Rs) principle (12).

Here, we integrated canonical withdrawal reflex-based behavioral assays with novel video-based voluntary assessments of NEP- (13) and movement-evoked pain- (MEP-) related behavior to bridge the translational gap. Using this battery of multidimensional behaviors, we characterized two widely used rodent pain models: the incision (INC) model (14), translating to postoperative pain, and the pathogen/adjuvant-induced inflammation (CFA) model representing inflammatory pain (15) to describe entity- and modality-specific behavioral trajectories in mice.

Furthermore, we investigated the role of the migration of Ly6G^+^ neutrophil granulocytes (NGs) in both pain entities and modalities using a comprehensive multidimensional test battery. NGs represent a significant immune cell population rapidly recruited after injury or during inflammation (neutropenia) (15). In addition to their contribution to wound healing and for fighting infection, NGs release pro-inflammatory cytokines [e.g., Interleukin-1β (IL-1β), complement factor 5a], various chemokines, growth factors, and endogenous opioids, all of which modulate the excitability of nociceptors (16). Therefore, it seems plausible that NGs are involved in the development of pain caused by a surgical incision or CFA, either by enhancing or ameliorating it. However, the role of NGs in evoked reflex-based, pain-related behavioral outcomes in both pain models is currently contradictory (15–17), and there are no studies on NEP- and MEP-related behavioral outcomes related to NGs to date. Thus, the aim of this study is to clarify an entity- and a modality-specific role of NGs in two rodent pain models using a comprehensive battery of pain-related outcome behaviors.

## Materials and Methods

The experiments in this study were reviewed and approved by the Animal Ethics Committee of the State Agency for Nature, Environment, and Consumer Protection North Rhine-Westphalia (LANUV, Recklinghausen, Germany), with the recommendations of the ARRIVE guidelines 2.0 (18) and were in accordance with the ethical guidelines for the investigation of experimental pain in conscious animals (19).

### General

**Figure 1:**
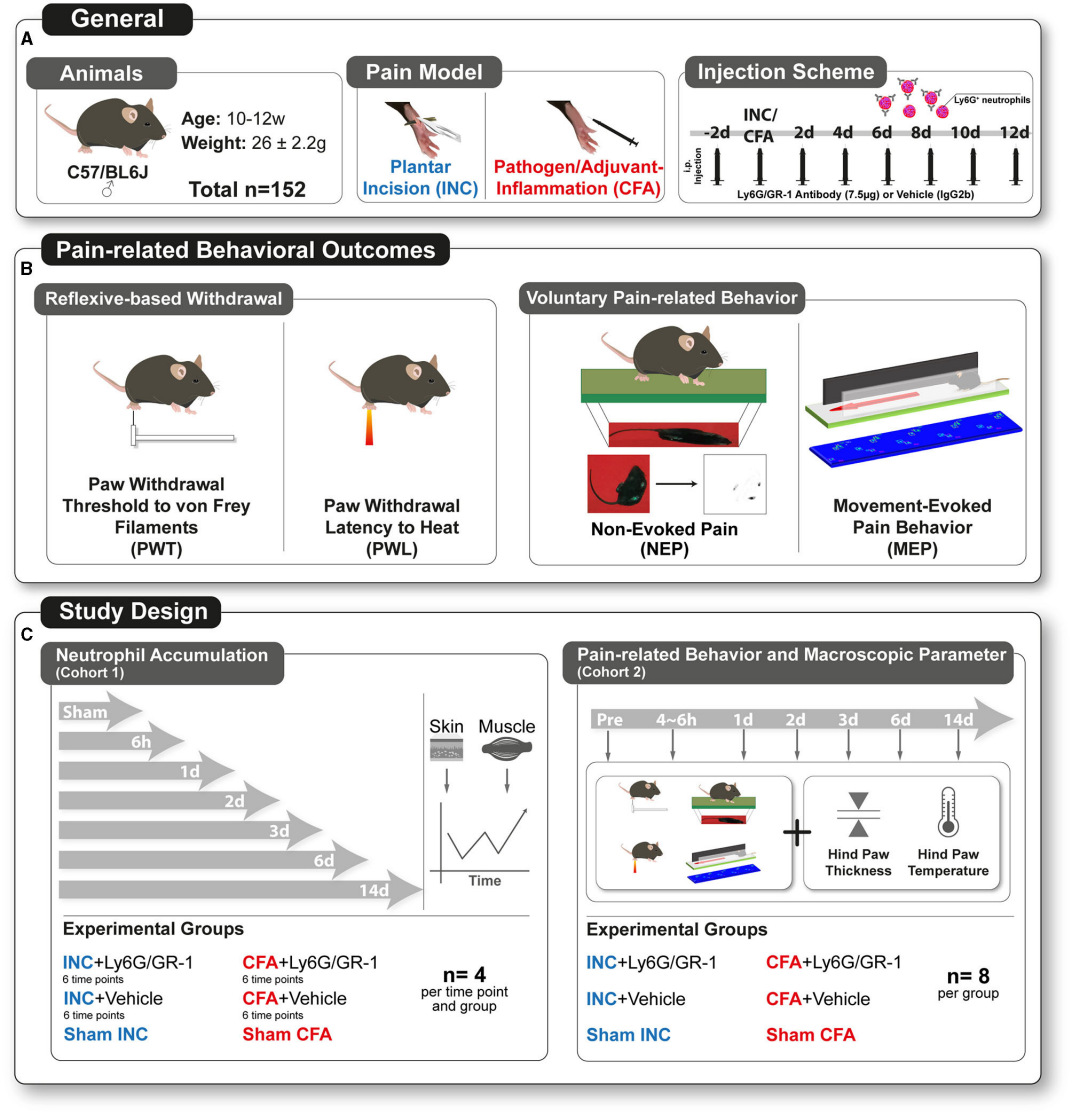
Study design. **(A)** Male C57/BL6J mice (age 10–12 weeks, weight 26 ± 2.2 g [mean ± SD)] were used. In this study, the plantar incision model (INC) as a surrogate for incisional pain, and the pathogen/adjuvant-induced inflammation (CFA) model as a surrogate for inflammatory pain were employed. To assess the role of Ly6G^+^ neutrophil granulocytes (NGs), mice were treated with intraperitoneal (i.p.) injection of Ly6G/GR-1 antibody (7.5 μg) or control antibody (IgG2b) every other day, starting 2 days before pain model induction (INC/CFA) to systemically deplete NGs. **(B)** Multidimensional pain-related behaviors were subgrouped into reflexive-based withdrawal, including punctate mechanical paw withdrawal threshold (PWT) to mechanical stimuli and paw withdrawal latency (PWL) to heat stimuli, and voluntary pain-related behavior, including non-evoked pain (NEP) and movement-evoked pain (MEP) behavior. **(C)** Mice of cohort 1 were deployed to study NGs accumulation in the skin and muscle of the plantar aspect of the right hindpaw at different time points. The characterization of the trajectories of multidimensional pain behavior and macroscopic parameters of the inflammatory response were assessed in a time-dependent manner with cohort 2. Mice from cohorts 1 and 2 were studied at different time points in relation to pain model induction. The neutrophil and antibody illustrations were created by modifying images purchased in the PPT Drawing Toolkits-BIOLOGY Bundle from Motifolio. All other illustrations were created by the authors themselves.

Adult male C57BL/6J mice [total *n* = 152 mice, 10–12 weeks, weight 26 ± (SD) 2.2 g] were kept in a 12/12 h day/night cycle with *ad libitum* access to food and water under standardized specific-pathogen-free (SPF) conditions (Figure 1A, General). According to their experimental group, mice were housed together (five per cage) to minimize intergroup effects. The experimental group allocation was random, and a blinded analysis of video-based behavior assessments was performed. Blinding to the withdrawal reflex-based behavioral assays and the pain model was not possible due to the testing conditions and visible signs of both pain models, for example, inflammatory reaction or sutures. Finally, animals were euthanized by an overdose of carbon dioxide at the end of the cohort-specific observation period.

### Pain Models: General Information

All procedures were performed on the right hindpaw of mice. Mice were initially anesthetized with 5% isoflurane in 100% oxygen; anesthesia was maintained with 1.5–2.0% isoflurane delivered through a nose cone during the whole procedure. No analgesics were administered.

### Surgery-Induced Postoperative Pain Model

Paw incision (Figure 1A, Pain Model) was based on the procedure introduced by Brennan et al. in rats and adapted to mice as previously described (14, 20). In brief, the entire right hindpaw was disinfected with 100% ethanol and Betadine® (povidone-iodine). No antibiotics were administered. On the plantar aspect, epidermis, dermis, fascia, and *musculus digitorum brevis* were incised longitudinally with a scalpel (No. 11, 0.5 cm). Additionally, the muscle was elevated and short-term retracted with forceps (Dumount #5) (21). A mattress suture of 7-0 Prolene within the skin was used to close the incision. The wound was covered by Betadine® using a cotton swab. The sutures were removed at the end of 2nd day under light isoflurane anesthesia. Sham-treated mice (anesthesia only, the same time duration) were used to control the surgical incision (Sham INC).

### Adjuvant/Pathogen-Induced Inflammatory Pain Model

As shown in previous studies, a CFA reaction was initiated by injecting 20 μl complete Freund's adjuvant (CFA, Sigma-Aldrich GmbH, Germany) subcutaneously (s.c.) into the plantar aspect of the right hindpaw with a 27-gauge Hamilton microsyringe (15) (Figure 1A, Pain Model). Sham-treated mice received an s.c. application of sterile 0.9% saline (20 μl) in the same aspect of the paw (Sham CFA).

### Systemic Depletion of NGs *via* Ly6G/GR-1 Antibody in Both Pain Models

Ly6G^+^ NGs were systemically depleted through an intraperitoneal (i.p.) injection of a purified anti-mouse Ly6G/GR-1 antibody (7.5 μg, RB6-8C5 clone, eBioscience, San Diego, CA, USA). Starting 2 days before the induction of pain models, the Ly6G/GR-1 or IgG2b (as vehicle control) antibodies were applied every other day throughout the entire experimental period (16) (Figure 1A, Injection Scheme) in both pain models. Untreated mice (no injection) were used as a separate control.

### Multidimensional Assessment of Pain-Related Behaviors

#### Reflex-Based Withdrawal Behaviors

##### Punctate Mechanical Paw Withdrawal Threshold

The punctate paw withdrawal threshold (PWT) was determined by the “percent response” method with a 60% withdrawal response (22, 23). Calibrated Semmes-Weinstein von Frey filaments in an ascending order (0.08, 0.2, 0.4, 0.7, 1.6, 4, 6, 10, 14, 20, and 40 mN) were applied five times per trial at a frequency of 1 Hz to the plantar aspect of the right hindpaw (Figure 1B). Mice were placed on a mesh grid, covered by a transparent plastic box (7 × 5 × 5 cm dimensions), and habituated for 15 min to the setting. The filaments were applied until the occurrence of 60% withdrawal response or reaching the cutoff limit of 40 mN. If so, 40 mN was regarded as PWT. The median force of three trials leading to a response was considered as the PWT to mechanical stimuli.

##### Paw Withdrawal Latency to Heat

The paw withdrawal latency (PWL) to heat was explored using a Hargreaves box (IITC Life Science Inc., Woodland Hills, CA, USA) (24). Here, mice were placed on a pre-warmed glass plate (30°C) in transparent plastic boxes (7 × 5 × 5 cm, dimensions) (Figure 1B). After 15 min of habituation, a radiant heat source was applied to the plantar aspect of the hindpaw. The intensity of the halogen lamp was adjusted to produce withdrawal latencies around 10–12 s at the baseline level (intensity:18%) (22). The latency to hindpaw withdrawal was measured with a cutoff time set to 20 s. Five trials at intervals of 5–10 min were performed to estimate the mean PWL to heat stimuli.

#### Voluntary Pain-Related Behaviors

##### Non-evoked Pain Assessment

Non-evoked pain was determined by comparing the weight-bearing (print area) of the affected (ipsilateral) with the non-affected paw (contralateral) at rest (Figure 1B) (13). Typically, INC and CFA caused an unbalanced weight-bearing distribution at rest due to guarding of the ipsilateral hindpaw. For this purpose, mice were separately placed in transparent boxes (7 × 5 × 5 cm, dimensions) on a 1-cm-thick and green light illuminated glass plate. Boxes were covered by a slim LED panel (illuminated in red) to enhance contrast. Without prior habituation, images of the footprints of mice were captured at intervals of 30 s for a total period of 10 min. The area of illuminated footprints of both hind paws was blindly determined on 10 different pictures for each mouse using ImageJ (25). The ratios of the illuminated ipsilateral to contralateral hindpaw area were calculated for each time point and averaged for every animal. The image selection was based on the predefined exclusion criteria, such as visible grooming, rearing, or an unsharp hind paw caused due to movement. The change in ratio represents the degree of guarding the affected hindpaw at rest.

##### Movement-Evoked Pain

Movement-evoked pain was assessed using the commercial CatWalk XT System (**Noldus** Information Technology, Wageningen, Netherlands) (Figure 1B). Only passed runs were included in the analysis and defined by a velocity range between 15 and 25 cm/s with a speed variance <60%. These inclusion criteria ensured comparability across all trials. Runs were recorded by a highspeed camera (100 fps), and the individual footprints were visualized by green light emitted into the glass plate on which the mice were running. Three passed runs were analyzed for each mouse and time point. Subsequently, each run was semiautomatically analyzed with the CatWalk XT software for two selected static (print area and stand duration) and dynamic (swing speed and stride length) gait parameters, which changed in different unilateral pain models (22, 26):

1. *Print area*: area of the whole paw
2. *Stand duration*: duration of ground contact for a single paw.
3. *Swing speed*: rate at which a paw is not in contact with the glass plate.
4. *Stride length*: length of one step.

### Assessment of Cardinal Inflammatory Signs

Inflammation is characterized by five cardinal signs: calor, tumor, rubor, dolor, and function laesa.

#### Calor: Hindpaw Temperature

A thermal imaging camera (Varioscan, InfraTec, Dresden, Germany) visualized the skin temperature of the ipsilateral hindpaw at each time point. Under short-term isoflurane anesthesia, animals were positioned with free access to the right hindpaw. Out of one image, the temperature of three different locations on the plantar aspect was measured using *Irbis Plus* (InfraTec, Dresden, Germany), leading to an average skin temperature of the ipsilateral hindpaw for each time point.

#### Tumor: Hindpaw Thickness

Following the determination of the hindpaw temperature, the dorsoventral thickness of the ipsilateral hindpaw was examined using a sliding caliper still under short isoflurane anesthesia. Three measurements were taken to estimate the dorsal-ventral paw thickness in millimeters at each time point.

### Study Design

#### Cohort 1: Analysis of Neutrophil Accumulation in the Traumatized Skin and Muscle of Ipsilateral Hindpaw

In cohort 1 (*n* = 104), the expression of myeloperoxidase (MPO), an NG-specific enzyme, was determined by Western blotting to assess NG accumulation in the skin (epidermis and dermis) and muscle biopsies sampled from the plantar aspect of mice ipsilateral to the incision/CFA injection on six different time points after model induction (Figure 1C, cohort 1, four mice per time point). Sham-treated groups (Sham INC and Sham CFA) were used as a control for basal expression of MPO in both tissues. After the pretreatment with lysis buffer, mechanical crushing, and sonification, proteins (quantified by the Bradford method) were denaturized by 10% SDS-polyacrylamide gel electrophoresis (Bio-Rad Laboratories, Hercules, CA, USA) and transferred onto nitrocellulose membranes (Whatman, Maidstone, UK, 0.45 μm, GE Healthcare, Chicago, IL, USA) by semi-dry blotting. Membranes were blocked with a skimmed milk solution (8%) and incubated with a primary antibody (1:3,000, Anti MPO antibody, 9535, Abcam, Cambridge, UK) at 4°C overnight. Subsequently, membranes were washed with Tris-buffered saline and Tween20 (TBST) for 15 min once and three times for 5 min. Next, the membranes were incubated with secondary antibody solution (1:5,000 NA 934, Amersham GE Healthcare, Amersham, UK) at room temperature for 1 h, followed by the same washing protocol. The housekeeping protein β-actin (1:20.000 β Actin Antibody, Sigma, St. Louis, MO, USA) was used as a loading control. The signals were visualized using an ECL system (Amersham GE Healthcare, Amersham, UK). The densitometric ratios of MPO signals were generated with the loading control.

#### Cohort 2: Behavioral Experiments and Macroscopic Parameters in Neutrophil-Depleted Mice and Controls After Incision and After CFA Injection

In cohort 2 (*n* = 48), mice injected with Ly6G/GR-1 antibody (*n* = 16) or IgG2b (*n* = 16) were assessed for behavioral phenotypes and macroscopic parameters in both pain models (Figure 1C, cohort 2). In sham-treated mice (no incision, no CFA injection, *n* = 16), no antibody was treated. Pain-related behaviors and macroscopic parameters were assessed before (pre) and on different time points (4–6 h, 1, 2, 3, 6, and 14 days) after incision and CFA injection (Figure 1C). All behavioral tests were performed on each mouse. The order of the behavioral tests was not randomized because of the potential influence of reflexive-based withdrawal tests on the outcome of voluntary behaviors. Therefore, we started first with voluntary tests (NEP and MEP), followed by the reflexive-based withdrawal tests (first PWT and second PWL). Finally, we measured the macroscopic signs of inflammation.

### Statistics

The results of the macroscopic parameters and MPO expression were analyzed by Kruskal–Wallis and Dunnett's multiple comparison tests. To achieve a better comparability of the individual behavioral data across the different pain modalities for this longitudinal study, mean changes in percent were calculated for NEP, PWT, and PWL (raw data are reported in Supplementary Figure 1). The ratiometric analysis of MEP expression is necessary as the weight of animals increases during the experimental period, and a comparison of the individual parameters on raw data is only possible to a limited extent. The Kruskal–Wallis and Mann–Whitney rank-sum tests were used for behavioral experiments followed by *post-hoc* tests (a two-tailed Dunnett's test). After the Friedman and Kruskal–Wallis test, multiple comparisons were performed by a Dunnett's test (to the sham group) for non-parametric raw data. Spearman coefficients (two-tailed) were used to determine relationships between the multiple parameters. Correlation analyses were performed after first computing the mean of side-by-side replicates and then analyzing those means. The value of *p* < 0.05 was accepted as significant. All results are expressed as mean ± SEM.

## Results

### Ly6G^+^ Neutrophil Accumulation and Effect of Depletion in INC and CFA Pain Models

**Figure 2:**
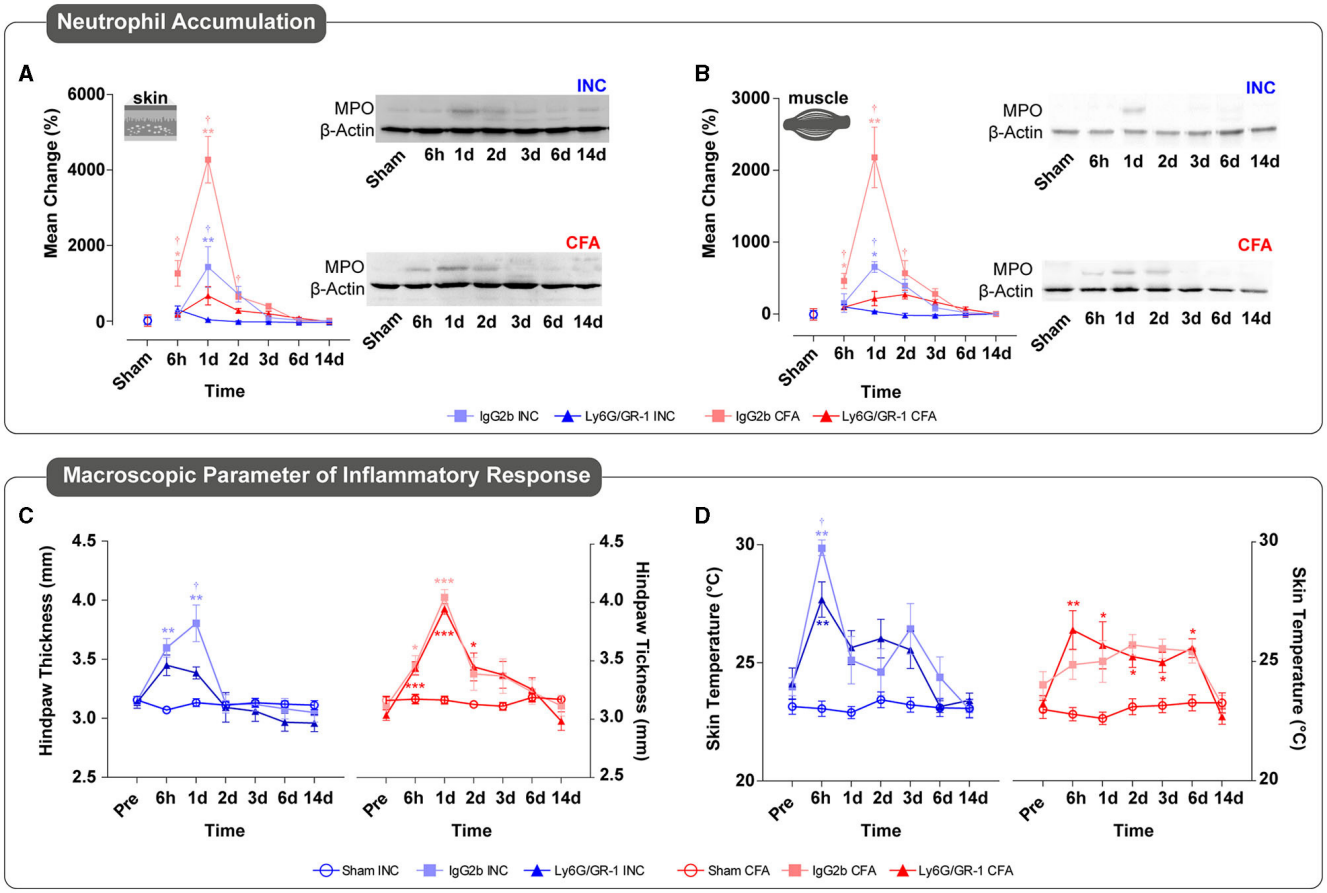
Time-dependent Ly6G^+^ neutrophil (NG) accumulation and macroscopic parameter of inflammatory response in incisional (INC) and inflammatory (CFA) pain models. **(A,B)** Upon INC and CFA, the NG-specific marker myeloperoxidase (MPO) was significantly increased in the skin **(A)** and muscle **(B)** tissue during the acute phase until day 2 after vehicle treatment in cohort 1. The treatment of Ly6G/GR-1 antibody significantly decreased MPO levels, thereby showing the absence of Ly6G^+^ neutrophils. All experimental groups contain four mice (cohort 1). The results are expressed as mean ± SEM. Values of *p*: ^*^p < 0.05, ^**^p < 0.01, ^***^p < 0.001 vs. Sham, ^†^*p* < 0.05, ^††^*p* < 0.01, ^†††^*p* < 0.001 vs. Vehicle (IgG2b) by the Kruskal–Wallis test followed by Dunnett's multiple comparison test. **(C,D)** INC and CFA induce a significant increase in the inflammatory response, represented by hindpaw thickness **(C)** and skin temperature **(D)** in cohort 2. While the inflammatory response with INC was limited to the acute period, CFA induced a longer-lasting response. Neutropenia induced by Ly6G/GR-1 treatment led to a significant decrease in both inflammatory parameters in the acute phase after incision, but no alterations were observed in the CFA group. All experimental groups contain eight mice (cohort 2). The results are expressed as mean ± SEM. Values of *p*: ^*^p < 0.05, ^**^p < 0.01, ^***^p < 0.001 vs. Pre-value, ^†^*p* < 0.05, ^††^*p* < 0.01, ^†††^*p* < 0.001 vs. Vehicle (IgG) at the same time point, by Kruskal–Wallis and Dunnett's multiple comparison tests.

To assess the Ly6G^+^ neutrophil accumulation and the efficacy of global depletion *via* Ly6G/GR-1 antibody, the expression of the NG-specific enzyme MPO was determined in both pain models in skin and muscle samples of ipsilateral hindpaws (cohort 1). In the IgG2b-treated (vehicle) group, MPO peaked significantly at 6 h and 1 day in CFA but only 1 day post-INC the skin (Figure 2A) and muscle (Figure 2B). Thereafter, progressive recovery of NG accumulation was observed in both pain models, which reached the baseline values 2 days post-injury. Ly6G/GR-1 antibody treatment significantly reduced MPO in both tissues and pain models (Figures 2A,B).

Edema formation and skin temperature of the ipsilateral hindpaw were determined over two weeks in cohort 2. In the acute period, ipsilateral hindpaw thickness increased significantly in both pain models under IgG2b control treatment with slightly different durations (until 1 day after INC and until 2 days after CFA injection) (Figure 2C). In IgG2b-treated mice, skin temperature of ipsilateral hindpaws increased significantly in INC mice in the acute phase but only until 6 h post-incision (Figure 2D). In contrast, skin temperature significantly increased for at least 6 days in the CFA group treated with the control antibody (Figure 2D). NG depletion using Ly6G/GR-1 antibodies significantly decreased the incision-induced thickness and temperature of ipsilateral hindpaws 6 h following the surgery relative to their vehicle controls. However, both parameters were not affected in NG-depleted CFA-treated animals (Figures 2C,D).

### Multidimensional Pain-Related Behavioral Outcome in INC and CFA Pain Models and the Role of Ly6G^+^ Neutrophils

**Figure 3:**
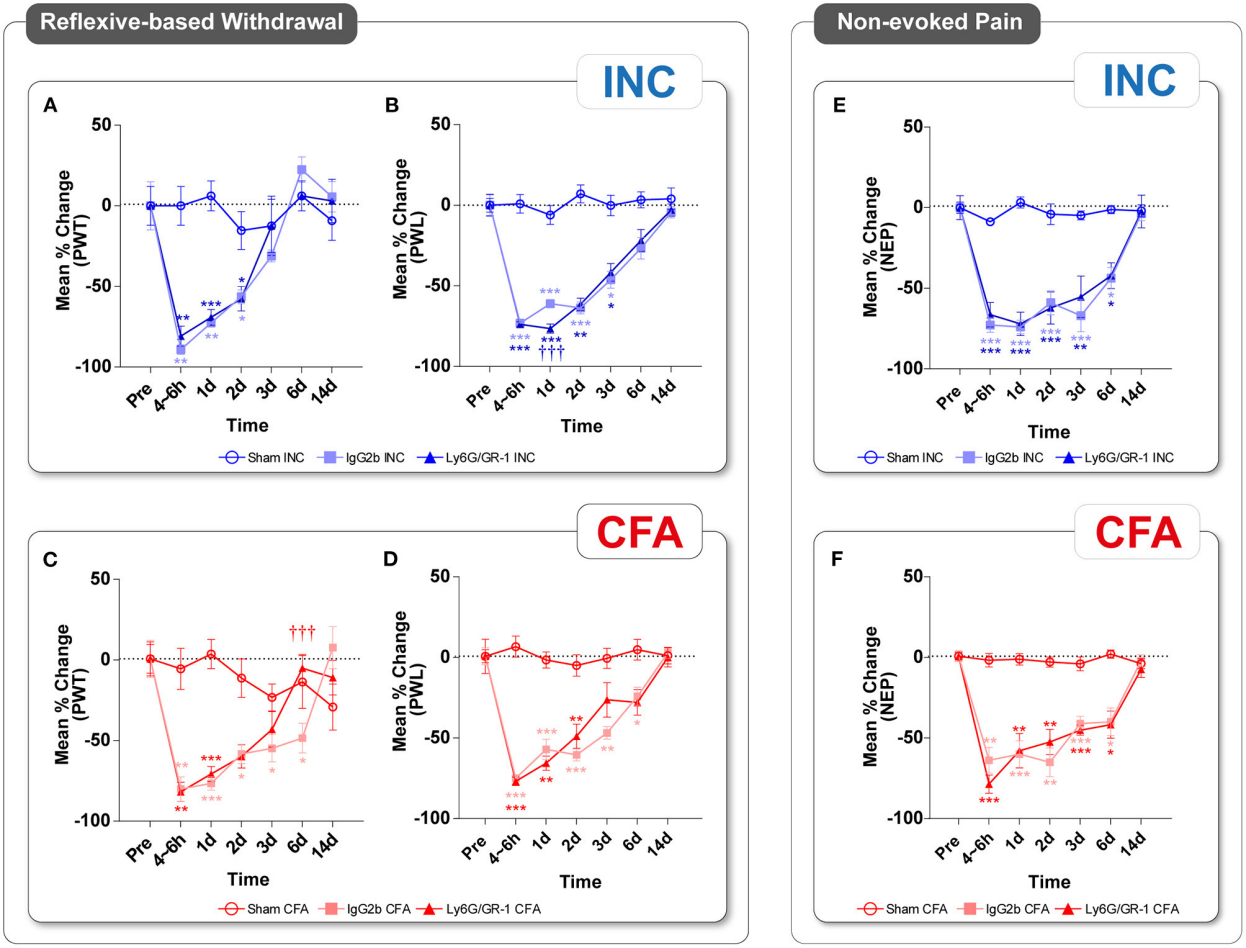
Multidimensional pain-related behavior after plantar incision or CFA. Trajectories of reflex-based withdrawal behavior **(A–D)**, including punctate mechanical PWT to mechanical stimuli and PWL to heat stimuli, and **(E,F)** NEP behavior, were determined in INC and CFA. PWT **(A,C)** and PWL **(B,D)** peaked at 4–6 h in both pain models but showed differences in their duration. NEP **(E,F)** was decreased in the acute phase and lasted up to day 6 in both pain entities. Ly6G/GR-1 antibody treatment attenuated PWT in CFA at day 6 and exacerbated PWL at 1 day in INC. Sham-mice showed no changes. All experimental groups contain **eight** mice (cohort 2). The results are expressed as mean ± SEM. Values of *p*: ^*^p < 0.05, ^**^p < 0.01, ^***^p < 0.001 vs. Pre, ^†^*p* < 0.05, ^††^*p* < 0.01, ^†††^*p* < 0.001 vs. Vehicle (IgG) by Kruskal–Wallis and Dunnett's multiple comparison tests.

Classical reflex-based withdrawal assays were used to determine the thresholds to mechanical stimuli (PWT) and the withdrawal latencies to heat stimuli (PWL) at the hind paw. In IgG2b-treated mice (vehicle group), PWT and PWL were significantly reduced by days 2 and 3 in INC animals, respectively (Figures 3A,B). PWT and PWL were significantly decreased by day 6 after CFA injection in IgG2b-treated mice (Figures 3C,D). INC and CFA sham-treated mice showed no changes in PWT and PWL (Figures 3A–D and Supplementary Figure 1). NG depletion slightly worsened PWL on day 1 after INC (^†††^*p* < 0.001), whereas PWT was unchanged in comparison to those injected with IgG2b. After CFA injection, the PWT in NG-depleted mice was only significantly decreased until day 2 in comparison to sham-treated mice and was significantly ameliorated at day 6 in comparison to IgG2b-control treatment. Heat hypersensitivity was ameliorated from day 3 after CFA injection compared to IgG2b-treated mice.

To move beyond classically evoked behavior assays, we employed a novel video-based assessment of non-evoked pain behavior (NEP) at rest. In this assay, NEP is represented by a decrease in the print area at rest of the ipsilateral paw. NEP was significantly reduced in both pain models starting in the acute phase (4–6 h) and remained significant until day 6 in the IgG2b-control mice group (Figures 3E,F). Ly6G^+^ neutrophil depletion did not modulate NEP in both pain models. Sham-treated mice showed no changes in NEP (Figures 3E,F and Supplementary Figure 1).

**Figure 4:**
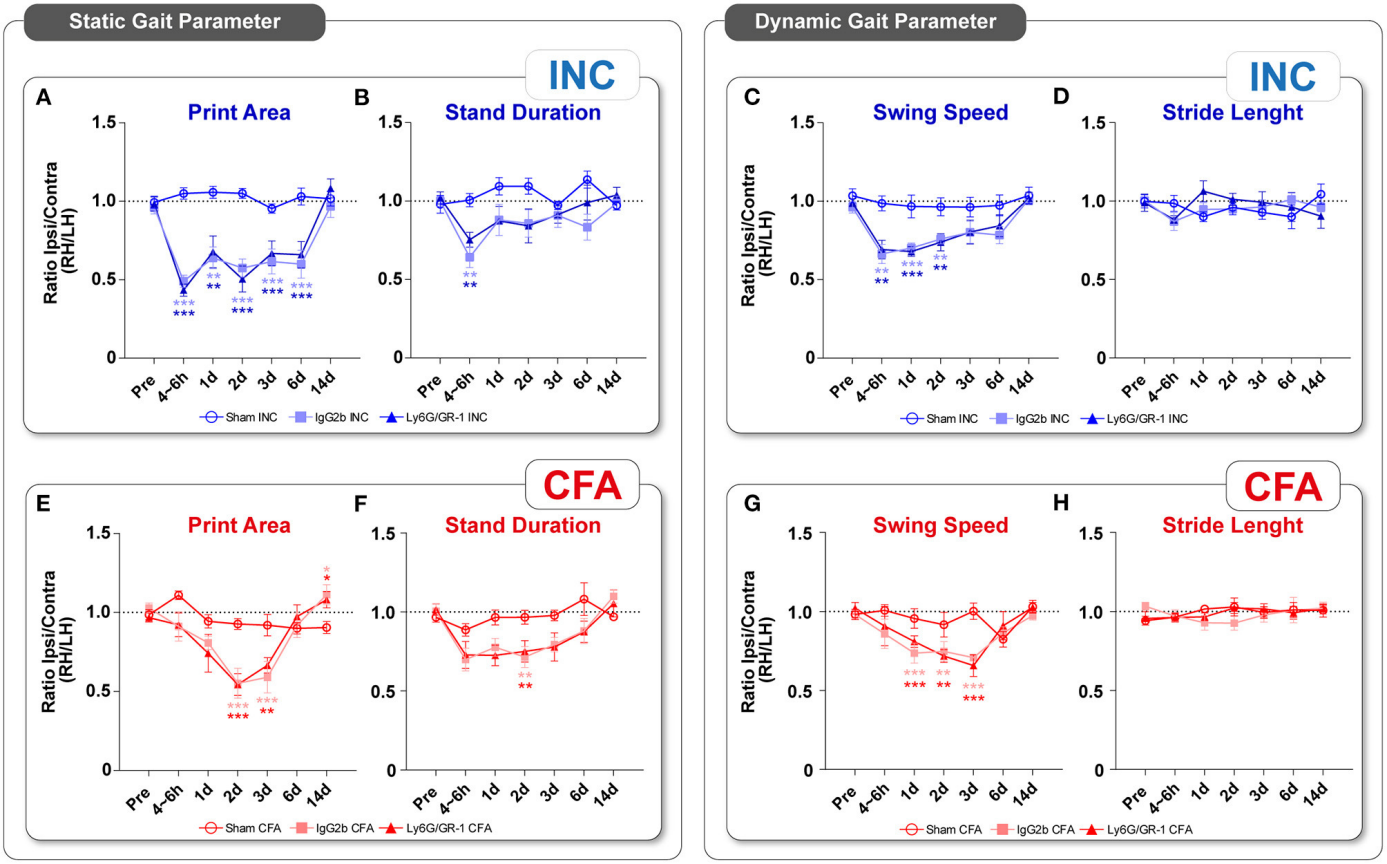
Antalgic gait after plantar incision or CFA. Upon INC, print area **(A)** was reduced until 6 days, whereas stand duration **(B)** and dynamic swing speed **(C)** were reduced in the acute phase after incision. Print area **(E)** was reduced in CFA at days 2, 3, and 14, whereas stand duration **(F)** and swing speed **(G)** were significantly altered at later time points. No changes were observed for the gait parameter stride length in both models **(D,H)**. Ly6G/GR-1-treated mice showed no differences to vehicle-treated groups, independent of pain model. No gait changes were observed in the Sham-mice. All experimental groups contain **eight** mice (cohort 2). The results are expressed as mean ± SEM. Values of *p*: ^*^p < 0.05, ^**^p < 0.01, ^***^p < 0.001 vs. Pre, ^†^*p* < 0.05, ^††^*p* < 0.01, ^†††^*p* < 0.001 vs. Vehicle (IgG) by Kruskal–Wallis and Dunnett's multiple comparison tests.

In addition to NEP, movement-evoked pain behavior (MEP) also represents a relevant symptom in different pain diseases. With the CatWalk XT system, static and dynamic parameters of voluntary ambulatory gait are evaluated under standardized conditions to assess MEP. Two static (print area and stand duration) and two dynamic (swing speed and stride length) gait parameters known to be altered in unilateral pain models were selected to assess MEP (Figure 4). Both static parameters and the dynamic parameter swing speed were altered significantly in the acute phase (4–6 h) post-incision in IgG2b-treated mice. However, in the further observation period, these profiles became diversified; the print area was decreased until day 6 (Figure 4A), stand duration returned to the baseline on day 1 (Figure 4B), and swing speed on day 3 after incision (Figure 4C). In addition, the stride length (Figure 4D) was not changed at any time point post-incision. In contrast to INC group, MEP was not detected in the acute phase after CFA (Figures 4E–H). The print area was significantly reduced at day 2 and 3, and stand duration only at day 3 (Figure 4E). Swing speed was significantly decreased from 1 to 3 days (Figure 4G). As in the INC group, the stride length was not modulated over time. For all pain-related gait parameters, baseline values were reached at day 14 at the latest in both models, except the print area in CFA mice. Ly6G^+^ neutrophil depletion did not significantly affect MEP in both pain models (Figure 4E).

### Correlation Analyses

**Figure 5:**
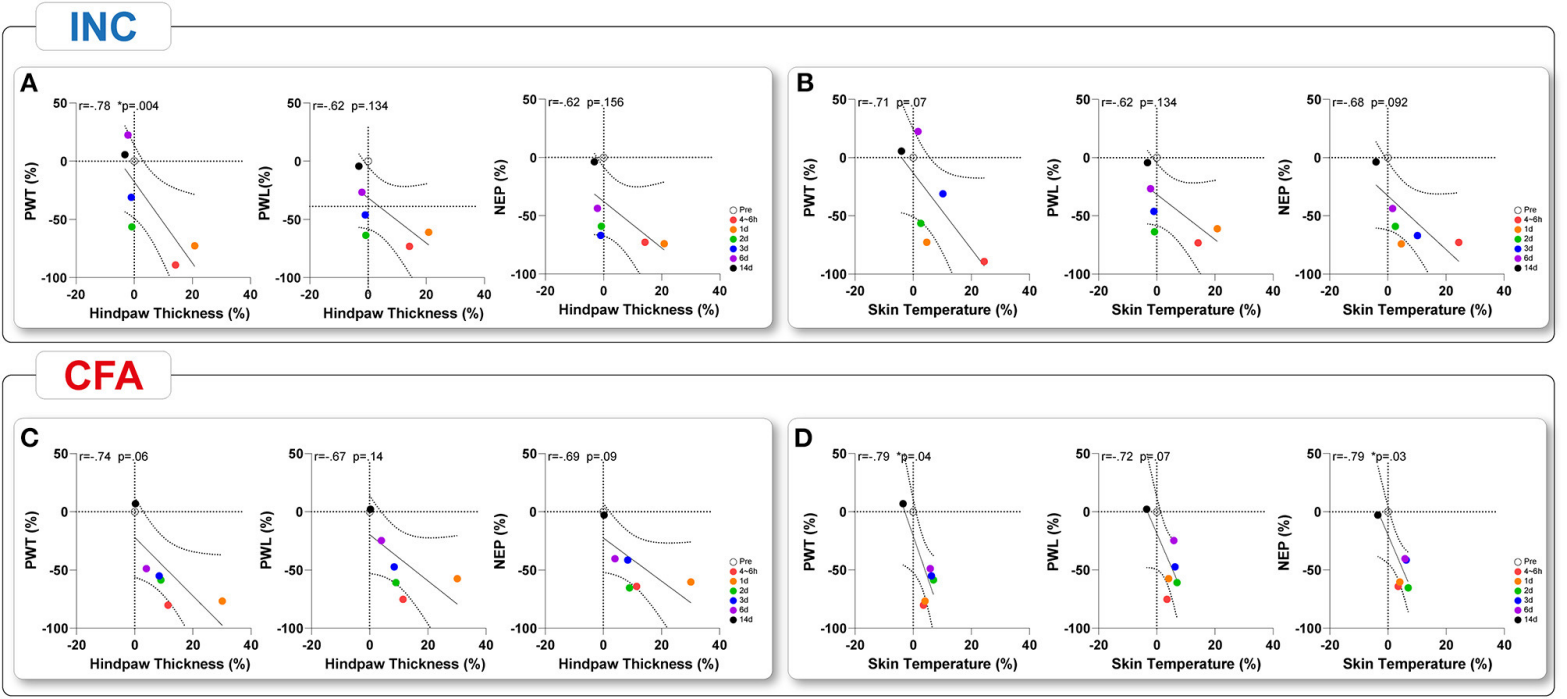
Time-dependent correlation analysis of the macroscopic inflammatory response parameter with reflexive-based withdrawal behavior to mechanical and heat stimuli and non-evoked behavior. **(A)** Upon INC, only the hindapw thickness was time-dependently correlated with PWT, **(B)** while hindpaw skin temperature did not show significant correlations. **(C)** No significant correlation were found between behavioral outcomes and hindpaw thickness in CFA mice but **(D)** between behavioral outcomes and skin temperatures. Linear regression (solid line) with 95% CI (dotted line) is displayed. Values of *p*: ^*^p < 0.05, ^**^p < 0.01, ^***^p < 0.001 by Spearman (two-tailed) coefficients were used to determine relationships between the multiple parameters. Correlation analyses were performed after first computing the mean of side-by-side replicates and then analyzing those means.

**Figure 6:**
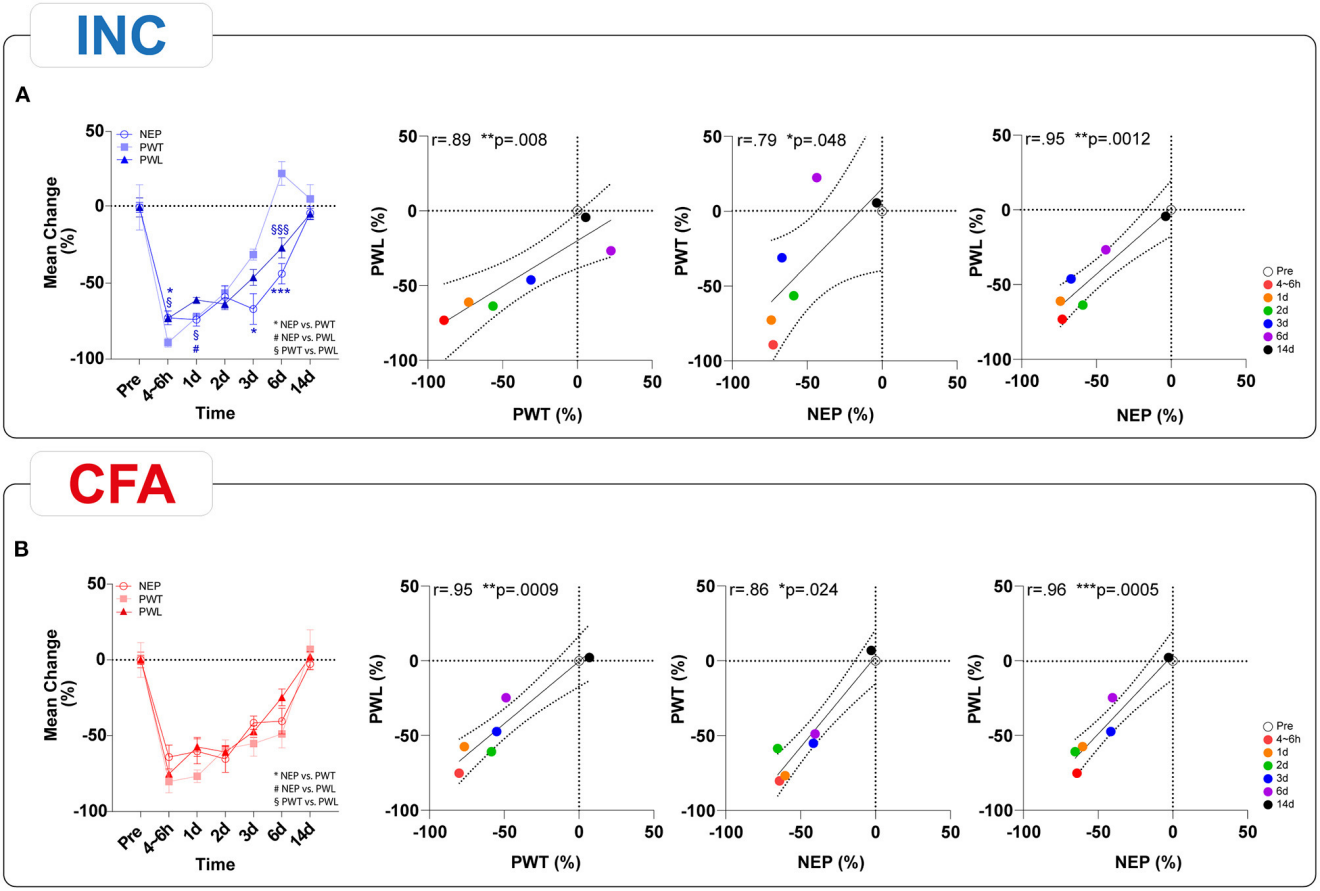
Time-dependent correlation analysis of NEP behavior assessment and withdrawal reflex-based behavioral assays in INC **(A)** and CFA **(B)** animals. In both pain models, trajectories of PWT, PWL, and NEP were significantly correlated. The results are expressed as mean ± SEM. Values of *p*: ^*^p < 0.05, ^**^p < 0.01, ^***^p < 0.001 NEP vs. PWT, ^#^*p* < 0.05, ^*##*^*p* < 0.01, ^*###*^*p* < 0.001 NEP vs. PWL, ^§^*p* < 0.05, ^§§^*p* < 0.01, ^§§§^*p* < 0.001 PWT vs. PWL, by Kruskal–Wallis and Dunnett's multiple comparison tests. Linear regression (solid line) with 95% CI (dotted line) is displayed. Values of *p*: ^*^p < 0.05, ^**^p < 0.01, ^***^p < 0.001 by Spearman (two-tailed) coefficients were used to determine relationships between multiple parameters. Correlation analyses were performed after first computing the mean of side-by-side replicates and then analyzing those means.

**Figure 7:**
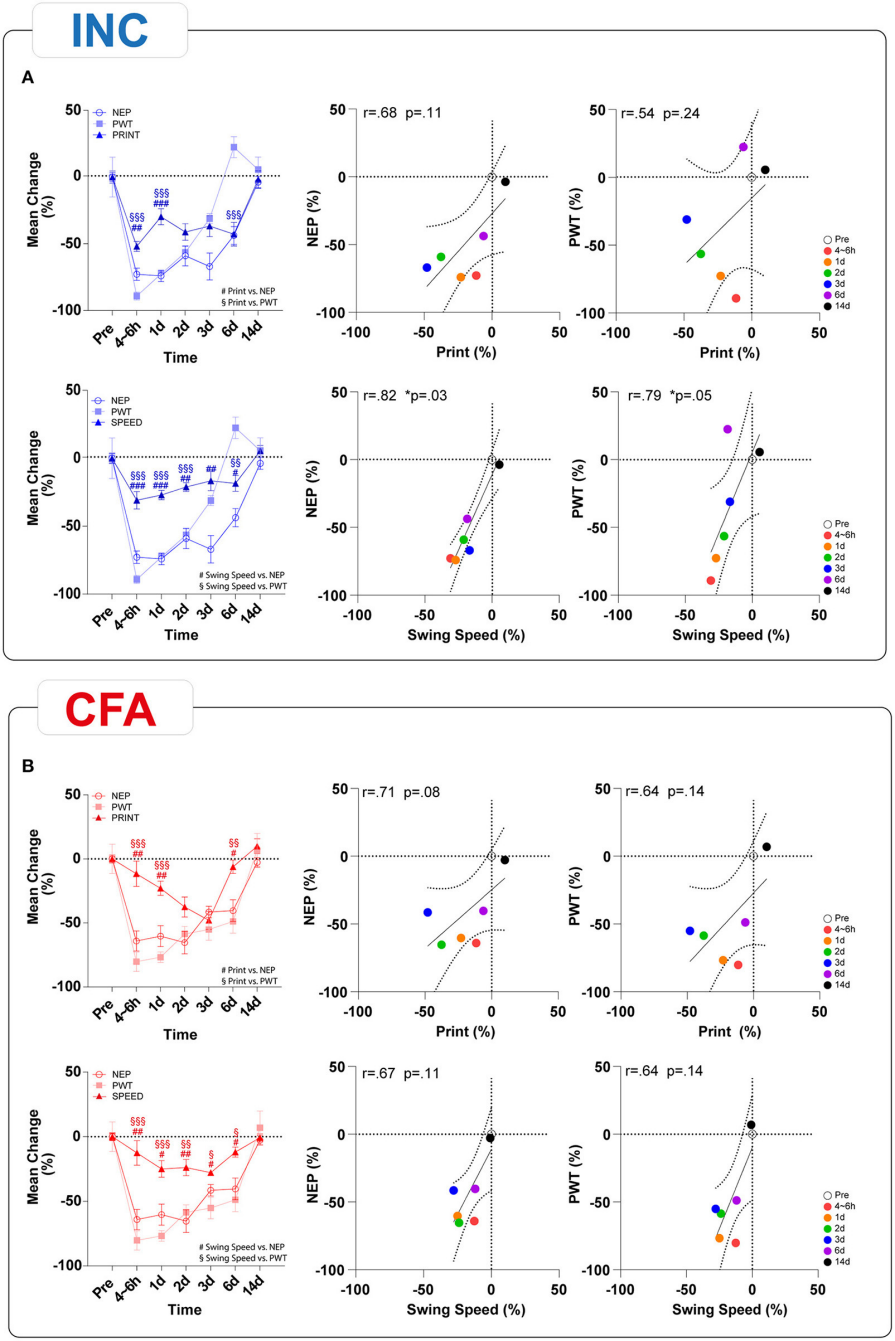
Time-dependent correlation analysis of static and dynamic gait parameters with NEP behavior and mechanical thresholds in INC **(A)** and CFA **(B)** animals. Upon INC, significant correlations were observed for the dynamic gait parameter “Swing Speed” with NEP and PWT. Neither the static parameter “Print” in the INC group nor all comparisons of CFA group, significant correlations could be found. Linear regression (solid line) with 95% CI (dotted line) is displayed. Values of *p*: ^*^p < 0.05, ^**^p < 0.01, ^***^p < 0.001 by Spearman (two-tailed) coefficients were used to determine relationships between multiple parameters. Correlation analyses were performed after first computing the mean of side-by-side replicates and then analyzing those means.

We performed a correlation analysis of behavioral outcome parameters to investigate the possible coherence between the multidimensional pain modalities that could inform about shared mechanistic pathways. Because Ly6G^+^ neutrophil depletion only showed marginal effects on pain-related behaviors in both pain models, we limited our analysis to mice receiving IgG2b. First, we performed a correlation analysis between macroscopic parameters and reflex-based withdrawal assessments to investigate a link between them (Figure 5). A significant time-dependent negative correlation was found across all time points only for PWT and hindpaw thickness for INC, indicating a decrease in PWT associated with an increase in edema formation (Figure 5A). Hindpaw skin temperature did not correlate with any behavioral tests in INC although we noted a trend (*p* = 0.07) for a negative correlation with PWT (Figure 5B). While there was only a trend (*p* = 0.06) between PWT and paw edema in CFA-treated mice (Figure 5C), a significant correlation between skin temperature and PWT was observed (Figure 5D). Further, NEP was negatively correlated with skin temperature in the CFA group (Figure 5D). Interestingly, responses to heat stimuli did not correlate with any inflammatory parameter. The correlation analysis between the reflex-based behavioral tests and the NEP showed significant positive correlations across all modalities and both pain models (Figure 6). When we correlated PWT and NEP with MEP parameters, a different picture emerged (Figure 7). Here, we found significant positive correlations of PWT and NEP with the dynamic parameter “swing speed” in the INC model. Other gait parameters did not show significant correlations.

## Discussion

Our comprehensive behavioral approach identifies behavioral outcome measures in two preclinical pain models in mice beyond reflex-based responses, which resemble clinical pain-related symptoms in patients. Time profiles of classical reflex-based withdrawal responses as the measures of mechanical (PWT) and heat (PWL) hypersensitivity correlate with NEP or differ from MEP, suggesting overlapping and distinct underlying mechanisms, respectively. Further, we show that the depletion of Ly6G^+^ neutrophils increased inflammatory responses in both pain models. However, pain-related behavioral responses were only slightly modified by NG depletion; here a short-term amplification of heat hypersensitivity after the incision and accelerated recovery of mechanical hypersensitivity in the inflammatory pain model are evident. Interestingly, the more complex multidimensional behavioral outcome measures were unaffected by NG depletion, indicating that Ly6G^+^ neutrophils might not play a major role in pain-related outcomes related to surgery and CFA.

### Multidimensional Pain-Related Behavior Is Transient and Represents Different Aspects of Incision and Pathogen/Adjuvant-Induced Inflammation Pain

The assessment of “pain” in rodents is a multidimensional approach and presently a matter of ongoing debate. Despite significant preclinical pain research efforts, a translational gap exists between pain-related outcomes in rodents and humans (1). This leads to an insufficient understanding of mechanisms of pain entities and the unsuccessful development of novel therapeutic options. To bridge the translational gap between preclinical and clinical pain measures (1, 6, 10, 27, 28), we used here novel video-based paradigms based on voluntary behaviors in mice to assess the behaviors that more likely resemble pain-related outcomes in patients. “Non-evoked pain” is a concept based on the modulations of various natural and rodent-specific behaviors like grooming, burrowing, weight-bearing, ultrasound vocalization, facial expression, emotional, and cognitive aspects, to name a few (10). It is also reflected by the scoring of the guarding behavior (21, 29, 30) and the spontaneous foot lifting (SFL) of the affected paw in unilateral pain models (31, 32). Problematic ones might be a scoring system prone to be biased by subjective rating. Thus, we developed a novel paradigm to detect guarding behavior as voluntary and more objectively by investigating the reduced print area of the injured hindpaw at rest in mice (13). Guarding is defined as “behavior […] aimed at preventing or alleviating pain” (33); as such, it could be a behavior to avoid mechanical stimulation as a result of contact with the ground or be a sign of ongoing pain or may be a mixture of both as described previously (33). Our results here demonstrate a significant guarding behavior based on this approach that peaked early after onset in both pain entities, lasted until day 6, and showed complete remission on day 14. The acute phase peak of guarding behavior was also described in the studies performed with subjective scoring in mice (21, 29) and rats (34). However, in contrast to rats, the determination of guarding behavior with the “scoring” assessment in mice is more difficult due to anatomical differences, higher mobility of mice, challenges in acclimatization to the experimental setting, and the impact of an observer (21). Therefore, only a few studies exist in the literature to describing non-evoked behaviors in mice in the incision (4, 21, 35) and the CFA model (36, 37), mainly due to methodological problems of this behavior assay. Using a video-based, standardized assay that can detect even small footprint-based area changes and underlies objective measures and no scoring, many disadvantages of the “scoring” method are eliminated, especially the experimenter bias. However, we have to admit that the time course of our footprint-based approach has a similar time course to the reflex-based pain behaviors; this suggests that the footprint approach might be at least a combination of non-evoked behavior as well as avoiding touch/pressure to the underlying ground. A pure “non-evoked” behavior that lacks the flaws of the scoring system after incision needs further investigation. Nevertheless, such an avoidance of behavior might as well be relevant for better translation to the clinics.

In line with previous findings in rats after plantar incision (38) and CFA-treated rodents (26), we were able to detect an antalgic gait, which is characterized by a decreased swing speed associated with a reduction of the stand duration and the print area of the hindpaw for both pain models early after onset injury. This type of movement-evoked behavior in mice might be of major importance to be studied in more detail. Recently, it was mutually agreed with patients and many stakeholders involved in perioperative pain management, pain-related physical function (like moving out of bed, performing physiotherapy, etc.) is the most important domain to be assessed in clinical acute perioperative pain (39). Future studies need to identify relevant mechanisms of movement-evoked pain in mice to develop effective treatment options.

A correlation analysis of pain modalities may inform about the entity- and modality-specific mechanisms. We show here a significant correlation between mechanical and heat hypersensitivity in both pain entities. According to previous studies, it is clear that mechanical and heat hypersensitivity underlie different pathophysiological mechanisms (40), these processes seem to correspond time-wise. Furthermore, both modalities were correlated with non-evoked pain behavior although the correlation was most pronounced with heat hypersensitivity. Further, movement-evoked behaviors and withdrawal reflex-based assessments do not correlate, suggesting that the underlying pathophysiological processes differ. These differences are already evident in the stimulus type; however, they highly suggest that movement-evoked mechanisms have been missed to be investigated in the past in preclinical studies. While reflexive paw withdrawal is evoked by punctual stimulation of distinct nociceptors/sensory types, the ambulatory activity of the hindlimb during movement reflects peripheral inputs that are the result of the integration of multiple tissues and nociceptor/sensory types throughout the paw and hindlimb. In addition, such a voluntary behavior might involve supraspinal responses and therefore was better related (than reflex-based approaches) to a more complex construct of pain in humans. Together, this might explain why translating results obtained with “old-school” behavioral assessments do not translate well to the clinic.

### Does a Modality-Specific Role of the Ly6G^+^ Neutrophils Exist for Incisional or Inflammatory Pain?

Currently, conflicting reports exist on whether pain-related behaviors are affected by antibody-mediated peripheral depletion of Ly6G^+^ neutrophils in C57BL/6 mice. While Carreira et al. demonstrated a reduction of mechanical hypersensitivity after Ly6G^+^ neutrophil depletion post-incision (17), others reported no change in mechanical thresholds accompanied by a slight worsening of heat hypersensitivity (16) or no effect on neither mechanical nor heat hypersensitivity after incision (15). In addition to these conflicting results, all findings have been based on reflex-based approaches only, reducing the generalizability and significance of the results.

Here, we show that Ly6G^+^ neutrophil depletion does not change mechanical hypersensitivity after incision but might have a transient, mild effect on incision-induced heat hypersensitivity. This is in contrast to the study of Carreira et al. who identified a major role of Ly6G^+^ neutrophils for mechanical hypersensitivity after incision (17). In addition, the results in the incision model differed from a previous study using the CFA model (15) where, in our hands, mechanical hypersensitivity was unchanged, but the recovery of heat hyperalgesia was accelerated. These conflicting results between the studies within the same pain model might be attributed to different timing and routes (i.v. vs. i.p.) of Ly6G/GR-1-antibody application as well as the differences in pain model induction characteristics and, as described above, bias in assessment. It is known that experimental parameters like incision depths are particularly crucial for the magnitude of immune response and relevant for nociceptor activation in dermis vs. muscle; thus, pain-model induction influences the outcome (41, 42). It is also noteworthy that edema and hypersensitivity do not necessarily correlate with incisional (16, 29) and inflammatory pain (29, 43). The different roles of Ly6G^+^ neutrophils for pain behavior in different pain models might be explained by a predominant secretion of either pro- or anti-inflammatory mediators in each pain model (16). This dichotomous role (anti- vs. pro-inflammatory) has been reported previously in different pain models (15, 44).

Antibody-based depletion of immune cells is a standard method to investigate their disease-specific role but is largely determined by antibody selectivity. To achieve maximum comparability with the previous studies investigating the role of Ly6G^+^ neutrophils in the context of pain in rodents, the most widely applied antibody (Ly6G/GR-1, clone RB6-8C5) was used in this study. However, this antibody may deplete, albeit marginally (15), various subtypes of monocytes and wound macrophages that have an impact on inflammatory processes and therefore must be considered when interpreting the data shown here (45).

Here, we show that Ly6G^+^ neutrophil recruitment plays a minor role in developing and maintaining hypersensitivity to mechanical and heat stimuli and is also done without any contribution to non-evoked and movement-evoked pain-related behavior associated with incisional and inflammatory pain. These findings are partially consistent with some reports highlighting the role of different immune cell types in both pain models that modulate sensitization nociceptors by releasing inflammatory mediators. However, as all previous studies were exclusively based on reflex-based behaviors, these results might not be of major relevance to be translated to the clinic. Indeed, due to the assessment of non-evoked and movement-evoked pain-related behavior with our multidimensional behavior approach, we clearly show that the migration of Ly6G^+^ neutrophils does not contribute to those pain-related behaviors, which translate to more clinically relevant outcomes in patients (e.g., physical function). Based on these data, peripheral Ly6G^+^ neutrophils in the skin and muscle tissue are not a promising cellular target for developing novel analgetic options in these two pain entities. In the future, a multidimensional behavioral approach lays the foundation for examining new and revisiting known mechanisms for their translational potential to gain novel perspectives, providing potential valuable information for translation to the clinic.

## Data Availability Statement

The original contributions presented in the study are included in the article/Supplementary Material, further inquiries can be directed to the corresponding author/s.

## Ethics Statement

The animal study was reviewed and approved by Animal Ethics Committee of the State Agency for Nature, Environment and Consumer Protection North Rhine-Westphalia (LANUV, Recklinghausen, Germany).

## Author Contributions

DS contributed to the study design, developed and performed behavioral experiments, determined macroscopic parameters, analyzed behavioral data, and created illustrations. BP contributed to the analysis of behavioral data and the preparation of illustrations. SR contributed to the study design and performed behavioral experiments. LCS determined macroscopic parameters and performed behavioral experiments. EP-Z initiated and designed the study and supervised the experiments and the data analysis. DS, BP, and EP-Z wrote the manuscript. All other authors reviewed the manuscript.

## Funding

This study was supported (in part) by the Interdisciplinary Center for Clinical Research (IZKF) Münster, Grant Pog2/027/20 to EP-Z.

## Conflict of Interest

In the last 5 years, EP-Z received financial support from Mundipharma GmbH and Grunenthal for research activities and from Grünenthal, MSD Sharp and DOHME GmbH, Mundipharma GmbH, Mundipharma International, Janssen-Cilag GmbH, Novartis AG, Fresenius Kabi, and AcelRx for advisory board activities and/or lecture fees. The remaining authors declare that the research was conducted in the absence of any commercial or financial relationships that could be construed as a potential conflict of interest.

## Publisher's Note

All claims expressed in this article are solely those of the authors and do not necessarily represent those of their affiliated organizations, or those of the publisher, the editors and the reviewers. Any product that may be evaluated in this article, or claim that may be made by its manufacturer, is not guaranteed or endorsed by the publisher.

## Supplementary Material

Supplemental Figure 1 Raw data of non-evoked pain (NEP) behavior assessment and withdrawal reflex-based behavioral assays in the plantar incision and pathogen/adjuvant-induced inflammation (CFA) pain model. Upon INC or CFA, pain-related behavior trajectories of NEP **(A,D)**, paw withdrawal threshold (PWT) **(B,E)**, and paw withdrawal latency (PWL) **(C,F)** hypersensitivity were determined. INC- (A) and CFA- **(D)** induced NEP started in the acute phase and lasted up to 6 days. Mechanical **(B,E)** and heat **(C,F)** hypersensitivity were peaked at 4–6 h in both pain models but were different in their duration. The attenuation of neutropenia by Ly6G/GR-1 antibody treatment significantly reduced mechanical hypersensitivity at day 6 in the CFA group and exacerbated heat hypersensitivity on day 1 in INC. Sham-mice showed no changes. All experimental groups contain **eight** mice. The results for NEP and PWL are expressed as mean ± SEM, PWT is displayed as median ± 95% CI. Values of *p*: ^***^*p* < 0.001 vs. Pre,^†^^†^^†^*p* < 0.001 vs. Vehicle (IgG) by Kruskal–Wallis and Dunnett's multiple comparison tests for GUB and PWL. Friedman and Dunnett's test was used for PWT.
