# Supplementary figures and images for: Investigating the Role of Ly6G+ Neutrophils in Incisional and Inflammatory Pain by Multidimensional Pain-Related Behavioral Assessments: Bridging the Translational Gap
Source: Front Pain Res (Lausanne). 2021 Sep 10;2:735838. doi: 10.3389/fpain.2021.735838 (PMC8915677; doi:10.3389/fpain.2021.735838)

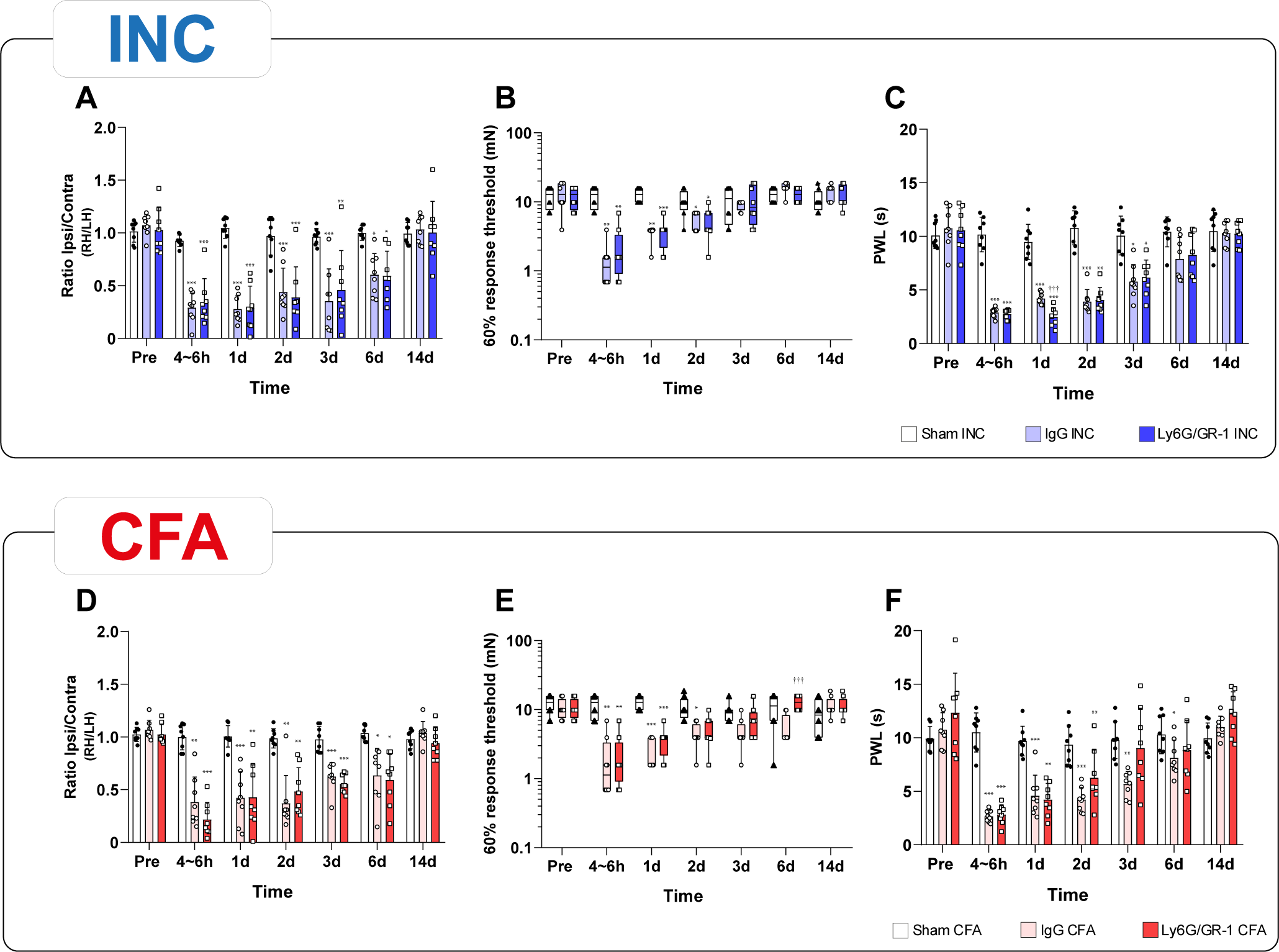

Supplement: Supplemental Figure 1 — Raw data of non-evoked pain (NEP) behavior assessment and withdrawal reflex-based behavioral assays in the plantar incision and pathogen/adjuvant-induced inflammation (CFA) pain model. Upon INC or CFA, pain-related behavior trajectories of NEP (A,D), paw withdrawal threshold (PWT) (B,E), and paw withdrawal latency (PWL) (C,F) hypersensitivity were determined. INC- (A) and CFA- (D) induced NEP started in the acute phase and lasted up to 6 days. Mechanical (B,E) and heat (C,F) hypersensitivity were peaked at 4–6 h in both pain models but were different in their duration. The attenuation of neutropenia by Ly6G/GR-1 antibody treatment significantly reduced mechanical hypersensitivity at day 6 in the CFA group and exacerbated heat hypersensitivity on day 1 in INC. Sham-mice showed no changes. All experimental groups contain eight mice. The results for NEP and PWL are expressed as mean ± SEM, PWT is displayed as median ± 95% CI. Values of p: ***p < 0.001 vs. Pre,†††p < 0.001 vs. Vehicle (IgG) by Kruskal–Wallis and Dunnett's multiple comparison tests for GUB and PWL. Friedman and Dunnett's test was used for PWT. [file Image_1.TIF]
